# Hormonal modulation, mitochondria and Alzheimer’s prevention: the role of GLP-1 agonists and estrogens

**DOI:** 10.3389/fmolb.2025.1622186

**Published:** 2025-06-26

**Authors:** Fernando Lizcano, Daniela Sanabria, Eliana Aviles

**Affiliations:** ^1^ Center of Biomedical Investigation (CIBUS), Universidad de La Sabana, Chía, Colombia; ^2^ School of Medicine, Universidad de La Sabana, Chía, Colombia; ^3^ Fundación Cardioinfantil-Instituto de Cardiología, Bogotá, Colombia; ^4^ School of Medicine, Universidad del Rosario, Bogotá, Colombia

**Keywords:** Alzheimerr′s disease, GLP-1 agonists, estrogens, prevention, metabolism

## Abstract

Alzheimer’s disease (AD) is the most prevalent cause of dementia worldwide, disproportionately affecting women and lacking effective disease-modifying therapies. While traditional approaches have focused on amyloid β (Aβ) plaques and tau pathology, emerging evidence highlights the role of metabolic dysfunction, mitochondrial impairment, and hormonal signaling in the pathogenesis of AD. Estrogens exert neuroprotective effects by modulating synaptic plasticity, enhancing mitochondrial bioenergetics, and reducing oxidative stress and inflammation. Similarly, glucagon-like peptide-1 receptor agonists (GLP-1RAs), initially developed for the treatment of type 2 diabetes, have demonstrated promising cognitive benefits, potentially mediated through improved insulin signaling, neuronal survival, and reduced β-amyloid (Aβ) and tau burden. This review explores the converging mechanisms through which estrogens and GLP-1RAs may act synergistically to prevent or delay the onset of AD. We examine the influence of sex differences in mitochondrial dynamics, estrogen receptor distribution, and GLP-1 signaling pathways, particularly within central nervous system regions implicated in AD. Preclinical studies using GLP-1-estrogen conjugates have shown enhanced metabolic and neuroprotective outcomes, accompanied by reduced systemic hormonal exposure, suggesting a viable therapeutic strategy. As the global prevalence of AD continues to rise, especially among postmenopausal women, dual agonism targeting estrogen and GLP-1 receptors may represent a novel, physiologically informed approach to prevention and intervention. Ongoing clinical trials and future research must consider sex-specific factors, receptor polymorphisms, and brain-region selectivity to optimize the translational potential of this combined strategy.

## 1 Introduction

Alzheimer’s disease (AD) represents the most prevalent form of dementia to date. According to the World Health Organization (WHO), over 55 million people worldwide live with some form of dementia, with approximately 10 million new cases reported each year. The prevalence of this condition varies by gender, with an estimated 8.1% of women and 5.4% of men over the age of 65 experiencing some form of dementia, nearly 70% of which corresponds to Alzheimer’s disease (AD) (Livingston et al., 2020; Montero-Odasso et al., 2020; OMS, 2021).

The etiology of AD is complex and multifactorial, characterized by progressive neuronal aging, synaptic loss, and dysfunction of neural networks (Knopman et al., 2021). Pathologically, AD is marked by the extracellular accumulation of β-amyloid (Aβ) plaques and intracellular neurofibrillary tangles composed of hyperphosphorylated tau (τ) protein (Shi et al., 2020). Aβ plaques originate from the aberrant processing of amyloid precursor protein (APP), mediated sequentially by β- and γ-secretases. This cleavage generates the membrane-bound C-terminal fragment CTF99, which is subsequently processed by γ-secretase to release Aβ peptides of 40–42 amino acids (Neha and Parvez, 2023). The aggregation of these peptides into oligomers and their subsequent deposition as extracellular senile plaques is facilitated by interactions with apolipoproteins and proteoglycans, contributing to the synaptic dysfunction and neurodegeneration that define the disease (Arjmand et al., 2024).

Additionally, the hyperphosphorylation of tau protein disrupts its binding to neuronal microtubules. Hyperphosphorylated tau induces the formation of insoluble protein aggregates and, ultimately, intracellular tangles. This abnormal accumulation interferes with axonal transport and promotes axonal degeneration (Kinney et al., 2018).

Therapeutic efforts aimed at neutralizing these pathological processes have thus far yielded limited results. Monoclonal antibodies targeting Aβ, such as aducanumab, lecanemab, and donanemab, have not demonstrated clinically meaningful efficacy (Terao and Kodama, 2024). Antibodies directed against tau protein, including semorinemab, tilavonemab, and gosuranemab, have shown unpromising preliminary results and fail to improve global patient functionality (Florian et al., 2023; Monteiro et al., 2023). In general, these treatments only modestly reduce disease progression, and their clinical application remains limited due to a lack of effectiveness (Livingston et al., 2020; Walsh et al., 2021; van Dyck et al., 2023).

For these reasons, recent investigations have expanded the focus to additional disease mechanisms. Notably, alterations in cholinergic signaling, neuroinflammation involving microglial activation, and calcium dysregulation have emerged as relevant contributors to AD pathogenesis. Moreover, increasing attention has been given to metabolic dysfunction, particularly in glucose metabolism and the bioenergetic role of estrogens, as potentially essential triggers of AD. Recent research has highlighted the therapeutic potential of glucagon-like peptide-1 receptor agonists (GLP-1RAs) beyond glucose metabolism, particularly in neurodegenerative diseases such as Alzheimer’s disease (AD). These agents have demonstrated neuroprotective effects, including the reduction of oxidative stress, enhancement of mitochondrial function, and attenuation of neuroinflammation. Furthermore, emerging evidence suggests that GLP-1RAs may modulate central insulin signaling and synaptic plasticity, pathways that are increasingly implicated in the pathophysiology of AD. In this context, exploring the mechanistic relationship between GLP-1 receptor activation and mitochondrial function in the brain may provide valuable insights into novel therapeutic strategies for AD (Liang et al., 2024).

This review highlights estrogens’ role in the central nervous system, with emphasis on their regulatory functions in mitochondrial metabolism. It also explores their potential as a pharmacological target for AD prevention.

## 2 Neuronal metabolism and Alzheimer’s disease

Neurons are subject to systemic metabolic regulatory processes involving carbohydrates and lipids. Metabolic disturbances that elevate cardiovascular risk also affect the cerebral microvasculature, contributing to cognitive decline and the development of dementia, including AD. Impaired cerebral perfusion compromises white matter integrity, deteriorates neural connectivity, and facilitates neurodegenerative processes (Kellar and Craft, 2020; Hernandez-Rodriguez et al., 2022).

The metabolic events most closely associated with AD include insulin resistance, hyperglycemia, lipid dysregulation, mitochondrial dysfunction, and oxidative stress. All these factors promote disease progression. While the exact mechanisms remain incompletely understood, type 2 diabetes mellitus (T2DM) is strongly linked to the pathogenesis of AD (Mittal and Katare, 2016). Both conditions share overlapping pathological mechanisms that impair cognitive function, eventually leading to Aβ deposition in the brain. Furthermore, chronic inflammation, oxidative stress, dyslipidemia, mitochondrial dysfunction, impaired insulin signaling, and synaptic dysfunction are all common features of T2DM and AD (Kapogiannis et al., 2019; Bernabe-Ortiz and Carrillo-Larco, 2022).

At the molecular level, central or peripheral insulin resistance can result from reduced insulin receptor expression, decreased binding affinity, and disruption of downstream signaling pathways (Tatar et al., 2003). Insulin exerts its cellular effects through two primary pathways: the mitogen-activated protein kinase (MAPK) pathway and the phosphatidylinositol 3-kinase (PI3K)-Akt pathway, the latter of which plays a crucial role in cell growth and survival. Insulin receptor activation triggers the recruitment of insulin receptor substrate (IRS) proteins, which, when phosphorylated on tyrosine residues, activate PI3K-Akt signaling. In contrast, serine phosphorylation of IRS proteins inhibits this signaling cascade (Craft and Watson, 2004).

The ratio of serine-phosphorylated IRS to total IRS is widely used as a biomarker of insulin resistance in both brain and peripheral tissues. An elevated ratio reflects greater insulin resistance. Studies have demonstrated cerebral insulin resistance in AD patients using *ex vivo* insulin stimulation and measurement of this ratio in brain tissue (Bassil et al., 2014).

Consequently, impaired insulin sensitivity reduces PI3K-Akt activation and downstream phosphorylation of proteins essential for neuronal survival, such as glycogen synthase kinase-3β (GSK3β). This promotes tau hyperphosphorylation and neurofibrillary tangle formation, hallmark features of AD. Additionally, defective insulin signaling contributes to neuronal energy deficits by decreasing glucose uptake and reducing the expression and function of glucose transporters GLUT3 and GLUT4 in the central nervous system (Schulingkamp et al., 2000).

Moreover, reduced cerebral vascularization observed in T2DM leads to chronic hypoxia, which is implicated in the progression of AD. Decreased oxygen supply disrupts neuronal energy homeostasis, induces oxidative stress, and compromises mitochondrial function (Neth and Craft, 2017). Under hypoxic conditions, cells activate adaptive responses mediated by hypoxia-inducible factor-1 (HIF-1), which regulates genes involved in angiogenesis, cell survival, and glucose metabolism. However, sustained HIF-1 activation in the context of neurodegeneration may exacerbate inflammation and promote Aβ production, thereby worsening synaptic dysfunction and neuronal damage (Zhang et al., 2007).

In addition to impaired glucose metabolism, extensive clinical, preclinical, and epidemiological data have linked lipid metabolic dysfunction to AD risk. Lipids are essential for numerous brain processes, including synaptic regulation, myelin sheath formation, and energy storage. Given the brain’s high lipid content, numerous studies have identified early alterations in specific lipid classes in AD, such as decreased plasmalogens, sulfatides, and elevated ceramides (Han, 2005). Lipids also modulate APP trafficking and processing, influencing neurotoxic Aβ peptide formation (Penke et al., 2018).

Apolipoprotein E (APOE) is a key lipid-transporting protein primarily involved in cholesterol and phospholipid metabolism in both peripheral tissues and the central nervous system. It exists in three major isoforms in humans -APOE2, APOE3, and APOE4- which differ by single amino acid substitutions and exhibit distinct structural and functional properties. While APOE3 is the most prevalent and considered the “neutral” isoform, APOE2 is often associated with protective effects against neurodegeneration. In contrast, APOE4 has been consistently linked to increased risk and earlier onset of Alzheimer’s disease (AD), possibly due to its detrimental influence on lipid homeostasis, mitochondrial integrity, and neuroinflammatory pathways (Volgman et al., 2024; Guo et al., 2025).

Apolipoprotein E4 (ApoE4) is a major genetic risk factor that connects lipid metabolism disorders with AD. Recent studies have highlighted lipid droplet accumulation in ApoE4 carriers, especially within phagocytic cells. In these microglia, lipid overload impairs phagocytosis and increases inflammatory responses, contributing to neurodegeneration. Lipid dyshomeostasis interacts with several AD pathogenic pathways, including amyloidogenesis, mitochondrial dysfunction, oxidative stress, neuroinflammation, and myelin degeneration (Yin, 2023; Jackson et al., 2024).

The human APOE gene encodes the 34-kDa lipid-binding protein ApoE, which mediates lipid transport throughout peripheral organs and between brain cells (Hauser et al., 2011). Compared to the common ε3 isoform, the ε4 variant is the strongest genetic risk factor for late-onset AD (Houlden et al., 1998), while the ε2 isoform significantly reduces risk (Reiman et al., 2020). Each ApoE4 allele increases AD risk three- to fourfold and lowers the age of onset by approximately 8 years (Neu et al., 2017).

ApoE4 also exerts pathological effects by disrupting lipid concentration homeostasis. ApoE4 carriers exhibit higher plasma levels of total cholesterol and triglycerides, but reduced HDL cholesterol, while ApoE2 carriers show the opposite pattern (Notkola et al., 1998; Huang and Mahley, 2014). Additionally, ApoE4 enhances cytosolic phospholipase A2 (cPLA2) activity, increasing arachidonic acid production. ApoE4-associated pathology can be mitigated by DHA-rich (docosahexaenoic acid) diets but worsens with high-cholesterol intake (Wang et al., 2005; Grimm et al., 2017).

Cholesterol, sphingolipids, and polyunsaturated fatty acids are particularly implicated in AD pathogenesis. Understanding lipid alterations may lead to therapeutic strategies targeting lipids, which could vary depending on disease stage, ApoE status, and metabolic profiles [28]. Other genes related to lipid metabolism and AD risk include TREM2, APOJ, PICALM, ABCA1, and ABCA7, all involved in lipid transport. Additionally, SREBP-2, a key regulator of cholesterol metabolism, has also been genetically associated with increased AD risk (Zhao et al., 2015; Kober and Brett, 2017; Shimano and Sato, 2017; Yin, 2023).

## 3 The mitochondrial theory of Alzheimer’s disease

Mitochondria possess their own genome, which enables the synthesis of proteins essential for their function. This genome encodes 13 subunits of the complexes that constitute the electron transport chain (ETC), while the remaining subunits, along with other mitochondrial proteins, are encoded by nuclear DNA. Due to the absence of histones, mitochondrial DNA (mtDNA) is particularly vulnerable to oxidative stress. Moreover, its limited capacity for repair and recombination increases the accumulation of mutations that compromise mitochondrial function (Elson et al., 2006). Structural alterations in mtDNA are exacerbated in patients with AD, with microscopic analyses revealing abnormally small mitochondria and disrupted cristae, particularly in the mammillary bodies and certain hypothalamic regions (Baloyannis et al., 2015; Baloyannis et al., 2016). Several studies have also reported a higher incidence of oxidized nucleotides and an increased number of mutations in the coding regions of mtDNA in AD patients (Wang et al., 2005).

Although little is known about the epigenetic regulation of mtDNA, significant differences in mtDNA methylation have been observed between individuals with AD and healthy controls (Stoccoro et al., 2017). Mitochondrial function depends on a dynamic equilibrium between fusion and fission. Mitochondrial fusion allows the merging of individual organelles, promoting the exchange of materials and dilution of damaged components, thus maintaining mitochondrial efficiency. In contrast, fission generates smaller mitochondria, facilitating the selective removal of dysfunctional organelles through mitophagy and ensuring proper mitochondrial distribution according to cellular energy demands.

In AD, increased mitochondrial fission leads to excessive fragmentation. Postmortem studies have shown elevated expression of mitochondrial fission proteins in the prefrontal cortex of AD patients (Manczak et al., 2011). Several experimental studies have demonstrated that mitochondrial dysfunction is an early and central feature in the pathogenesis AD. In rodent models, intracerebroventricular (icv) administration of streptozotocin (STZ) has been widely used to mimic sporadic AD by inducing brain insulin resistance, oxidative stress, and cognitive decline, without affecting peripheral glycemic control. Unlike systemic administration, which causes selective pancreatic β-cell destruction via GLUT2 transporters and is used to model type 1 diabetes mellitus (T1DM), icv-STZ acts directly on neurons in the central nervous system (CNS). Notably, STZ-treated animals exhibit alterations in mitochondrial dynamics, including increased expression of fission proteins such as Drp1 and Fis1, accompanied by decreased levels of fusion-related proteins like Mfn2 and OPA1, particularly in the hippocampus and in the prefrontal cortex (Paidi et al., 2015; Joshi et al., 2018). Transgenic AD models such as APP/PS1 mice, which overexpress mutant human amyloid precursor protein and presenilin-1, have also revealed mitochondrial fragmentation and respiratory deficits, reinforcing the hypothesis that impaired mitochondrial dynamics and mitophagy are mechanistically linked to AD pathology. These findings suggest that mitochondrial fragmentation and bioenergetic failure contribute to neurodegeneration and cognitive impairment in this model.

Given that mitochondrial bioenergetic alterations appear early in the disease course, they are considered potential primary events underlying synaptic failure, neuroinflammation, oxidative stress, and neuronal loss (Joshi et al., 2018). Mitochondria are central to ATP production via oxidative phosphorylation and regulate calcium homeostasis, cell growth, and metabolism (Du et al., 2010; Peggion et al., 2024).

One hallmark of AD is the impaired removal of damaged mitochondria due to defective autophagy. A particular alteration involves lysosomal dysfunction, which hinders the degradation of structurally damaged mitochondria, leading to cellular toxicity (McGill Percy et al., 2025). Furthermore, mitochondrial impairment can, in turn, disrupt endolysosomal processes, as endolysosomal biogenesis may be modulated in response to mitochondrial damage (Fernandez-Mosquera et al., 2017). Studies have shown that mitochondrial dysfunction alters lysosomal function and morphology, either through exposure to mitochondrial toxins or deletion of proteins such as apoptosis-inducing factor (AIF), PTEN-induced kinase 1 (PINK1), or the ubiquitin ligase Parkin (Demers-Lamarche et al., 2016).

In AD, dysfunctional mitochondria accumulate and exacerbate lysosomal degradation bottlenecks. Affected neurons exhibit mitochondrial membrane potential loss, resulting in microtubule network disintegration and impaired autophagic flux toward lysosomes (Silva et al., 2017; Brewer et al., 2020). Aβ-induced oxidative stress further disrupts mitochondrial mobility and function (Brewer et al., 2020), establishing a negative feedback loop wherein Aβ exacerbates mitochondrial dysfunction, which in turn promotes further Aβ accumulation.

Mitochondrial dysfunction also contributes to tau pathology by increasing τ oligomer levels and shifting the monomer–oligomer balance toward toxic oligomers (Weidling et al., 2020). During oxidative phosphorylation, mitochondria generate and scavenge reactive oxygen species (ROS). However, persistent ROS overproduction exceeding antioxidant capacity leads to damage of cellular macromolecules, including phospholipids, proteins, and nucleic acids, compromising cellular function (Lane C. A. et al., 2018).

Elevated ROS levels can also result from Aβ modifications; for instance, the Met35 residue of Aβ, along with upregulated oxidases such as NADPH oxidases and monoamine oxidase B (MAO-B), increase ROS production. Moreover, Aβ interactions with excess metals-such as Fe^2+^, Cu^2+^, and Zn^2+^-further elevate oxidative stress (Lane D. J. R. et al., 2018; Bai et al., 2022).

## 4 Role of estrogens in neuronal function

Estrogens are steroid hormones primarily produced in the ovaries of women of reproductive age. Their primary biological activity is mediated by receptors belonging to the nuclear receptor superfamily, which function as transcription factors that modulate gene expression. After crossing the plasma membrane-facilitated by their steroid structure-estrogens bind to cytoplasmic receptors, which translocate to the nucleus and interact with DNA to regulate transcription (Lonard and O'Malley, 2006).

Estrogen receptors exist in two main isoforms, ERα and ERβ, both widely distributed throughout the body (Matthews and Gustafsson, 2003). These receptors form dimers and bind to specific DNA sequences known as estrogen response elements (EREs) in the promoter regions of target genes. Notably, approximately one-third of estrogen-regulated genes lack canonical EREs, suggesting alternative regulatory mechanisms (Johri et al., 2024; Peralta and Lizcano, 2024). Molecular and biochemical studies have shown that estrogens may also exert transcriptional effects via protein–protein interactions with other transcription factors (Aranda and Pascual, 2001; Marino et al., 2006; Mauvais-Jarvis et al., 2013).

Many estrogenic effects are not genomic and occur more rapidly than expected from transcriptional activation. The discovery of the G-protein–coupled estrogen receptor (GPER1) in the early 2000s provided insight into these non-genomic mechanisms (Nilsson et al., 2011). GPER1 mediates rapid signaling cascades involving adenylate cyclase, cyclic AMP, protein kinase A, and other second messengers. GPER1 mRNA and protein have been detected in blood vessels and cardiac tissue across various species (Haas et al., 2009). Expression in adipocytes, hepatocytes, and myocytes is more variable, and the precise *in vivo* role of GPER1 remains under debate (Meyer et al., 2011; Hewitt et al., 2017; Luo and Liu, 2020).

**FIGURE 1:**
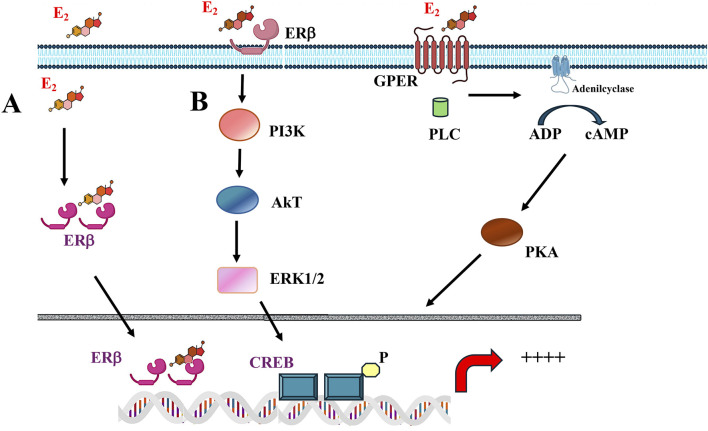
Estrogen signaling mechanisms. **(A)** Genomic signaling: Estrogens cross the plasma membrane and bind to the cytoplasm’s estrogen receptors (ERs). The estrogen-ER complex moves into the nucleus, forming homodimer and/or heterodimer complexes. These complexes bind to specific estrogen-sensitive elements (EREs) in DNA or recruit transcription factors. **(B)** Non-genomic signaling: Estrogens can perform a non-genomic effect by binding to their receptors on the plasma membrane. Additionally, some ERb are in the plasma membrane that induce the signaling cascade. Non-genomic information is established by extracellular signaling that stimulates second messengers in the cytoplasm; Responses are mediated by specific G-protein-coupled (GPER) receptors or estrogen receptors on the membrane. Finally, non-genomic action can indirectly increase gene expression.

Ligand-independent actions of estrogen receptors (ERs) have also been described, particularly in the uterus, where ERα can be activated by growth factor pathways (e.g., IGF-1), leading to receptor recruitment to chromatin in the absence of estrogen binding (Meyer et al., 2011; Hewitt et al., 2017) (see Figure 1).

Estrogens play a multifaceted role in the central nervous system (CNS), influencing synaptic plasticity, neuronal development, and survival in newly formed spinal synapses, as well as promoting neural stem cell proliferation and maintaining the integrity of the blood-brain barrier (McCullough et al., 2003; Mukai et al., 2010; Frick et al., 2015; Na et al., 2015). Both neuron- and astrocyte-derived estrogens are believed to contribute significantly to neuroprotection and cognitive function through interactions with ERs expressed in multiple brain regions (McEwen and Woolley, 1994; McEwen et al., 1995; Azcoitia et al., 2001).

Preclinical and human studies have shown that ERα is abundantly expressed in the hypothalamus, particularly in the preoptic area (POA), ventromedial nuclei (VMN), amygdala, and periventricular nuclei (PV). ERβ exhibits a similar distribution, maintaining high expression in the POA, bed nucleus of the stria terminalis (BNST), PV, and supraoptic nuclei (Laflamme et al., 1998; Azcoitia et al., 2001; Kruijver et al., 2003; Mitra et al., 2003; Kelly and Ronnekleiv, 2012). ERβ is also expressed in the hippocampus, amygdala, and cerebral cortex, where it participates in adult neurogenesis, synaptic plasticity, and new neuron formation (Weiser et al., 2008).

Postnatal expression of ERβ tends to decline, but it remains present in microglia, oligodendrocytes, and specific brain regions, such as the hypothalamus and amygdala (Vargas et al., 2016). ERβ signaling has been associated with several potential therapeutic benefits in CNS disorders: (1) it enhances GABAergic over glutamatergic signaling, exerting anticonvulsant effects (Veliskova and Desantis, 2013); (2) promotes oligodendrocyte maturation and myelination (Vargas et al., 2016; Karim et al., 2018); (3) modulates microglial activation and reduces inflammation (Valdes-Sustaita et al., 2021); and (4) supports serotonergic neurons, providing antidepressant effects (Suzuki et al., 2006). In both rodent models and postmenopausal women, ERβ ligands have shown beneficial effects on anxiety, depression, epilepsy, and multiple sclerosis (Warner and Gustafsson, 2015; Jellinger, 2024). However, clinical outcomes in humans have been inconsistent, potentially due to alternative splicing variants of ERβ, which may alter therapeutic responsiveness (Kim et al., 2018; Ulhaq and Garcia, 2021). Some natural compounds with estrogenic effects, such as polyphenols, have a beneficial impact on neurons by mitigating oxidative stress, inflammation, and apoptosis. However, their direct effects on the progression of Alzheimer’s disease have not been established (Abdelsalam et al., 2023).

Estrogens can also be synthesized *de novo* in the brain by neurons and astrocytes, starting from cholesterol (Blakemore and Naftolin, 2016). Moreover, local steroid metabolism in the brain can produce estrogens via aromatase, the enzyme responsible for converting androgens into estrogens (Gillies and McArthur, 2010; Brann et al., 2022). Aromatase expression varies across brain regions, with high levels found in the cerebellum, amygdala, hippocampus, and white matter. While sex differences in expression are not apparent in these regions, elevated aromatase levels have been reported in the hypothalamus—specifically in the POA and VMN—of male animals, suggesting regulation by circulating testosterone, which is subsequently converted to estradiol (E2) (Gillies and McArthur, 2010).

## 5 Estrogen effects on mitochondrial function

It is also important to consider the impact of estrogens on mitochondrial function. Mitochondria play a critical role in regulating cell survival and apoptosis, and the respiratory chain is a principal structural and functional target of estrogenic activity. Estrogens exert protective effects against oxidative stress by promoting the translocation of specific cytosolic enzymes into mitochondria, thereby shielding mitochondrial DNA (mtDNA) from free radical-induced damage (Leclere et al., 2013; Arjmand et al., 2024).

The distribution of ERα and ERβ in patients with AD varies considerably across brain regions. In women with AD, increased ERα expression has been observed in certain hippocampal areas, whereas levels are lower in hypothalamic nuclei and the medial mammillary nucleus (Hestiantoro and Swaab, 2004). However, overall, ERα has not been consistently implicated in AD pathogenesis. In contrast, growing evidence supports a protective role for ERβ, which appears to influence both disease risk and progression. In animal models, ERβ overexpression has been associated with reduced Aβ plaque deposition. Human studies have reported decreased ERβ levels in the frontal cortex of women with AD (Long et al., 2012; Tian et al., 2013).

**FIGURE 2:**
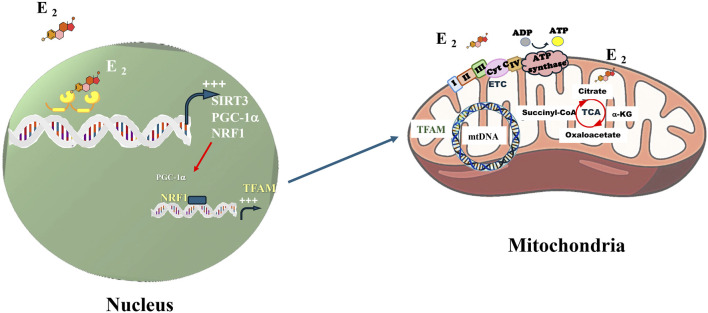
Los estrógenos pueden inducir la expresión de genes que aumentan la función mitocondrial, como SIRT3, PGC-1α, NRF1. ERβ increases NRF-1 which, in turn, increases TFAM that stimulates mtDNA transcription. Estradiol can increase glucose utilization by cells and ETC activity and prevent ROS production. E2 regulate many enzymes in the TCA. E2, estradiol; mtDNA, mitochondrial DNA; SIRT3, Sirtuin 3; PGC-1α, Peroxisome proliferator-activated receptor gamma, coactivator-1 alpha; NRF1, Nuclear respiratory factor −1; ERβ, Estrogen receptor beta; TFAM, Transcription factor A, mitochondrial; TCA, tricarboxylic acid cycle; ETC, Electron transport chain.

The presence of estrogen receptors within mitochondria was initially identified in MCF-7 breast cancer cell lines and later confirmed in brain cells. Estrogens may influence mitochondrial function through both direct and indirect mechanisms. Specifically, ERβ upregulates nuclear respiratory factor 1 (NRF-1), which in turn stimulates the expression of mitochondrial biogenesis regulators, including mitochondrial transcription factor A (TFAM), and multiple subunits of the mitochondrial respiratory chain (MRC). These factors contribute to the regulation of mitochondrial gene expression and the maintenance of mitochondrial homeostasis (see Figure 2).

A decline in estradiol (E2) levels leads to significant reductions in mitochondrial function, resulting in oxidative stress and impaired cerebral bioenergetics. Preclinical studies have shown increased hippocampal Aβ accumulation under E2-deficient conditions. This phenotype is characterized by reduced maximal respiratory capacity, diminished basal oxygen consumption, and lower extracellular acidification rates—indicative of decreased lactate production and glycolytic activity (Irwin et al., 2012).

Estrogens regulate the expression and activity of key enzymes in glycolysis, including hexokinase, phosphoglucoisomerase, phosphofructokinase, aldolase, glyceraldehyde-3-phosphate dehydrogenase, phosphoglycerate kinase, 6-phosphofructo-2-kinase, and fructose 2,6-bisphosphatase (Lizcano and Guzman, 2014). Estrogens also enhance the expression of glucose transporters GLUT3 and GLUT4 in the brain (Stirone et al., 2005; Razmara et al., 2008). In addition, estrogenic regulation extends to enzymes of the tricarboxylic acid (TCA) cycle, such as citrate synthase, mitochondrial aconitase 2, isocitrate dehydrogenase, and succinate dehydrogenase (Nilsen et al., 2007; Alaynick, 2008; Lizcano, 2022).

## 6 Do GLP-1 receptor agonists influence mitochondria and Alzheimer’s disease?

Type 2 diabetes mellitus (T2DM) and AD share metabolic abnormalities, including insulin resistance, mitochondrial dysfunction, inflammation, and increased oxidative stress. Incretin-based antidiabetic therapies, such as glucagon-like peptide-1 receptor agonists (GLP-1RAs), may offer benefits for individuals at risk of neurodegeneration due to their central effects on appetite and satiety regulation via the hypothalamus (Correia et al., 2012). Beyond glucose lowering and weight reduction, GLP-1RAs have shown positive effects on cognitive dysfunction in T2DM patients (Reich and Holscher, 2022).

While GLP-1 is primarily synthesized and secreted by intestinal L-cells in response to nutrient intake, it is also produced in the brain, particularly in the nucleus of the solitary tract in the brainstem. Following the ingestion of carbohydrates and fats, GLP-1 is released and exerts multiple physiological effects essential for maintaining glucose homeostasis (Bullock et al., 1996). It promotes glucose-dependent insulin secretion from pancreatic β-cells, ensuring proportional release relative to glycemia, and concurrently inhibits glucagon secretion from α-cells, reducing hepatic glucose production. GLP-1 also delays gastric emptying, thereby moderating postprandial glycemic excursions, and enhances satiety via central nervous system (CNS) signaling, contributing to appetite suppression and weight loss (Drucker, 2006).

**FIGURE 3:**
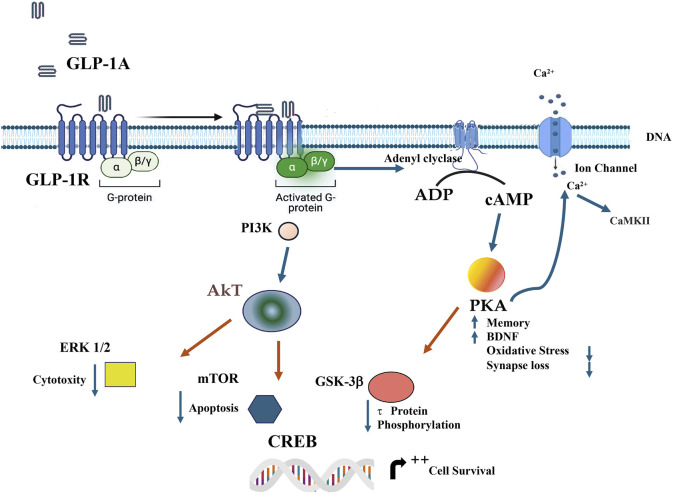
GLP-1RA activation initiates a cascade of signaling events through G_αs_ coupling, leading to the activation of adenylyl cyclase (AC) and subsequent production of cAMP. Protein kinase A (PKA) acts as a central mediator of downstream effects, modulating various ion channels. These channels jointly regulate membrane depolarization and calcium (Ca^2+^) influx. Parallel signaling through Akt activates ERK 1/2, which differentiates cellular responses: nuclear translocation of ERK 1/2 leads to transcriptional activation via mTOR and CREB, while cytoplasmic activation targets specific partners. The PI3K/AKT pathway regulates cell survival and metabolism. Calcium-dependent activation of CaMKII modulates these processes, emphasizing the complexity and versatility of GLP-1R signaling. Activation of PKA increase Memory, BDNF reduce oxidative stress and synapse loss. GSK-3β reduce tau phosphorylation, Akt reduce cytotoxity and apoptosis. PI3K, phosphoinositide 3-kinase; PKA, Protein kinase A; AkT, protein kinase B; ERK, Extracellular Signal-regulated Kinase; mTOR, mammalian target of rapamycin; GSK-3β, glycogen synthase kinase-3β; CREB, cAMP response element-binding protein; BDNF, Brain-derived neurotrophic factor; CaMKII, Ca2+/calmodulin-dependent protein kinase II.

Metabolically, GLP-1 acts through G protein-coupled receptors to improve insulin sensitivity in muscle and adipose tissues while enhancing β-cell function by promoting proliferation and inhibiting apoptosis (see Figure 3). Additionally, GLP-1RAs exert cardiovascular benefits, including improved endothelial function, reduced blood pressure, and cardioprotection. Clinically, GLP-1RAs—such as exenatide, liraglutide, dulaglutide, semaglutide, and tirzepatide—are used to manage T2DM, mimicking endogenous GLP-1 activity. These agents not only enhance glycemic control but also promote satiety and delay gastric emptying, making them effective in treating obesity (Drucker et al., 1987; Kreymann et al., 1987; Mojsov et al., 1987; Turton et al., 1996; Baggio and Drucker, 2007). The effects of GLP-1 on different tissues have demonstrated an enhanced insulin effect on skeletal muscle through SESN2-mediated autophagy and the attenuation of IRS1 serine phosphorylation (Tian et al., 2023). In obese mouse models, liraglutide improved insulin sensitivity in visceral adipose tissue, which was associated with reduced endoplasmic reticulum stress and increased Akt phosphorylation following insulin stimulation (Jiang et al., 2018). However, regarding the proliferation of insulin-producing pancreatic beta cells, there is no consensus regarding the ability to stimulate cell proliferation. Although GLP-1 receptor agonists have been shown to induce β-cell proliferation in rodent models, particularly in young animals, this effect has not been consistently replicated in human islets, where β-cell replication capacity is markedly limited. Thus, in humans, GLP-1 action appears to be predominantly functional and anti-apoptotic rather than proliferative (Dai et al., 2017; Buteau et al., 2003).

Both peripherally secreted GLP-1 and pharmacologic GLP-1RAs can cross the blood–brain barrier (BBB) or interact with circumventricular organs, suggesting that peripheral administration is sufficient to reach CNS targets. Central administration of GLP-1RAs reduces food intake, likely via GLP-1 receptor–expressing regions in the hypothalamus and brainstem (Kastin and Akerstrom, 2003), many of which are also estrogen targets (Kanoski et al., 2011), supporting a potential interaction between these systems (Miller, 2012). Substantial evidence suggests that GLP-1R signaling in the central nervous system (CNS) modulates reward-driven behavior, including food cravings and substance addiction, underscoring the broader implications of these peptides in neurobehavioral regulation (Shirazi et al., 2013; Richard et al., 2015).

Recent investigations have explored the therapeutic potential of GLP-1 in cardiovascular disease, metabolic-associated fatty liver disease, Parkinson’s disease, and AD. These multifaceted mechanisms underscore the relevance of GLP-1 in managing metabolic, cardiovascular, and neurodegenerative disorders (Cukierman-Yaffe et al., 2020; Arredouani, 2025).

The cloning and characterization of the GLP-1 receptor (GLP-1R) marked a key milestone in understanding GLP-1’s mechanisms of action. Pharmacological and molecular biology studies identified GLP-1R as a high-affinity receptor expressed across diverse tissues. As a member of the G protein–coupled receptor (GPCR) family, GLP-1R features a large extracellular domain responsible for ligand recognition and binding (Drucker and Holst, 2023).

The widespread expression of GLP-1R suggests that GLP-1 plays pleiotropic physiological roles beyond insulin secretion. In the CNS, GLP-1 has demonstrated neuroprotective effects, particularly in the hippocampus and cerebral cortex—key regions for memory and learning. These benefits include reducing neuroinflammation, improving neuronal energy homeostasis, and limiting neurodegenerative progression (Adams et al., 2018; Chang et al., 2018).

Potential mechanisms by which GLP-1RAs enhance cognition in T2DM patients include attenuation of oxidative stress, suppression of neuroinflammation, inhibition of apoptosis, reduction or prevention of Aβ accumulation, and mitigation of tau aggregation (Yaribeygi et al., 2021). Several preclinical studies have confirmed the ability of GLP-1RAs to reduce Aβ and tau deposits. Although findings in human studies have been less consistent, a pilot study reported reduced cerebrospinal fluid levels of Aβ42, and a large-scale clinical trial is currently underway to assess the efficacy of a GLP-1RA in early-stage AD patients (Crook and Edison, 2024; Cummings et al., 2025).

These pharmacological advances have reshaped the management of metabolic diseases, improving glycemic control, weight reduction, and cardiovascular outcomes. As research continues, GLP-1’s therapeutic potential in AD remains a promising frontier (Gejl et al., 2016; Du et al., 2022). Phase II clinical trial results with liraglutide support this hypothesis. In a multicenter UK study involving 204 participants randomized to liraglutide or placebo, cognitive decline in the liraglutide group was 18% slower than in the placebo group after 1 year of treatment (Femminella et al., 2019). Liraglutide may reduce neuroinflammation, decrease insulin resistance, improve neuronal communication, and limit Aβ and tau pathology.

Currently, the first randomized, double-blind phase III trial is underway to evaluate the effects of oral Semaglutide in AD prevention and progression. The EVOKE and EVOKE + trials are supported by robust preclinical evidence demonstrating GLP-1RA benefits in neurodegeneration and cognitive enhancement in T2DM patients (Akimoto et al., 2020; Cukierman-Yaffe et al., 2020; Norgaard et al., 2022).

## 7 Could dual agonism of estrogen and GLP-1 influence Alzheimer’s disease prevention?

The social and clinical impact of GLP-1RA therapy is reflected in the growing number of individuals using these medications. Currently, one in eight U.S. adults over the age of 18 reports having used a GLP-1 analogue (Harris, 2024). Additionally, novel combination therapies—such as Tirzepatide, which combines a GLP-1 analogue with glucose-dependent insulinotropic polypeptide (GIP)-are rapidly gaining traction, and it is expected that new formulations will continue to emerge, incorporating peptides capable of modulating metabolism and thermogenesis (Jastreboff et al., 2022; Rosenstock et al., 2023; Loomba et al., 2024).

Despite their widespread use, certain aspects of GLP-1 function, particularly its actions in the central nervous system (CNS), remain poorly understood—an important gap given the growing number of patients receiving these treatments. A key limitation of preclinical research, including GLP-1RA studies, is that most experiments are conducted in male animals. This introduces variability in pharmacodynamics, as GLP-1RA penetration into specific brain regions may differ based on sex, compound properties, and age (Borchers and Skibicka, 2025).

This raises important questions for ongoing trials such as EVOKE, which may uncover sex-specific differences and justify subsequent investigations of GLP-1 analogues in female populations. Moreover, the combined effect of GLP-1RAs with other molecules may vary depending on sex. Known polymorphisms and splice variants in ERβ could influence the efficacy of estrogen therapy in AD and may also impact GLP-1RA activity. Exploring these potential interactions could enhance our understanding of brain-targeted pharmacotherapy (Holscher, 2018; Jastreboff et al., 2022).

Sex differences in the incidence and progression of aging-related neurodegenerative diseases, particularly Alzheimer’s disease, are well established and globally consistent. In the United States, although men have higher age-adjusted mortality rates for 8 of the 10 leading causes of death, AD is a notable exception: women experience higher prevalence and AD-specific mortality, making it the only major cause of death more common in women (Budreviciute et al., 2020). This trend extends beyond the U.S., with higher AD burden reported among women in Europe, the United Kingdom, Japan, and other regions (Benziger et al., 2016).

Today, nearly two-thirds of patients receiving medical care for AD are women. This statistic reflects both a higher age-adjusted incidence of AD and longer life expectancy among women (Report, 2024). However, this female predominance appears paradoxical given the greater prevalence of classical AD risk factors-such as cardiovascular and metabolic diseases-among men. One explanation involves survival bias: men with high AD risk may die earlier from cardiovascular events, precluding the clinical manifestation of dementia. Sociocultural factors, such as unequal access to education in the 20th century (a known protective factor), may also play a role.

From a biological standpoint, several hypotheses have been proposed to explain female susceptibility to AD. Mitochondrial dysfunction plays a central role in AD pathophysiology, but sex differences in mitochondrial function remain poorly defined. Experimental models have shown that male and female animals differ in mitochondrial substrate utilization under stress conditions, though findings vary by context. For instance, in 3xTg-AD mice, sex-specific alterations in brain bioenergetics have been observed: females showed impaired complex I activity in synaptic cortical mitochondria, while non-synaptic mitochondria exhibited enhanced complex II–mediated respiration (Stojakovic et al., 2021).

In developmental neurobiology, Arnold proposed three categories of sex differentiation mechanisms: (1) organizational differences driven by fetal hormonal exposure; (2) activational differences induced by the adult hormonal environment; and (3) genetic differences linked to chromosomal content (Arnold, 2022). Studies using transgenic hAPP mouse models have shown that XY animals exhibit more severe clinical courses and earlier mortality compared to XX animals, independent of gonadal sex. This suggests a protective role of the second X chromosome (Davis et al., 2020), reinforcing the need to consider both genetic and hormonal factors when designing targeted therapies, including those involving hormonal agonists and estrogens for AD prevention (Lopez-Lee et al., 2024).

The beneficial effects of estrogens and GLP-1 agonists observed in other metabolic diseases raise the possibility that their combined use could also prevent or delay the onset of Alzheimer’s disease. A dual GLP-1-estrogen conjugate (GE) designed to selectively deliver estrogen to GLP-1R + cells induced substantial weight loss in mice without evidence of systemic estrogenic effects, as assessed by uterine weight and growth of estrogen-dependent breast cancer xenografts (Finan et al., 2012). Genetic studies in mice suggested that these effects were mediated by CNS GLP-1Rs, with increased expression of POMC and leptin receptors in the arcuate nucleus (Kanoski et al., 2011). Selective estrogen delivery to β-cells via a GE conjugate improved viability in both human and murine β-cells, again without systemic estrogen exposure (Sachs et al., 2020).

In another preclinical study, GE demonstrated superior metabolic effects compared to GLP-1-GIP or GLP-1-GIP-glucagon multiagonist therapies in models of polycystic ovary syndrome (PCOS), a condition frequently associated with obesity and insulin resistance. Chronic GE administration in female mice with PCOS significantly improved metabolic profiles, outperforming individual agonists. In the PWA model, GE suppressed hypothalamic expression of BCAP31, a pro-apoptotic protein, and promoted proteins associated with autophagy-critical processes for neuronal function and energy homeostasis. Altered CAMKII expression, which regulates orexigenic neuropeptides like NPY, also reflected compensatory adaptation to weight loss (Sanchez-Garrido et al., 2024).

Previous studies in obese rodents and diet-induced obesity models have shown that GE’s primary site of action is central, particularly within hypothalamic regions such as the supramammillary nucleus, lateral hypothalamus, and the nucleus of the solitary tract (Tiano et al., 2015). This therapeutic strategy-combining GLP-1 and estrogen receptor activation—was developed to enhance metabolic benefits while limiting systemic estrogen exposure, thereby minimizing reproductive and oncogenic risks. Data confirm that this approach enables tissue-specific action in GLP–1R–expressing regions, avoiding adverse effects in reproductive organs (Vogel et al., 2016).

Proteomic analyses of the hypothalamus following GE treatment revealed downregulation of inflammation-, apoptosis-, and immune-related proteins, and upregulation of pathways related to autophagy, vesicular transport, and intracellular signaling. These changes may help restore central energy homeostasis and explain the marked weight loss observed even at moderate GE doses (Schwenk et al., 2015).

## 8 Conclusion

Alzheimer’s disease remains a complex neurodegenerative disorder with multifactorial origins, including amyloid accumulation, tau pathology, insulin resistance, mitochondrial dysfunction, and chronic inflammation. The evidence reviewed herein underscores the critical role of estrogens in maintaining neuronal homeostasis, particularly through their effects on mitochondrial efficiency, antioxidant defense, and synaptic resilience. In parallel, GLP-1 receptor agonists have demonstrated neuroprotective actions beyond their established metabolic benefits, offering a promising avenue for cognitive preservation in at-risk individuals.

The convergence of estrogen and GLP-1 signaling on metabolic and neuroinflammatory pathways supports the rationale for dual-targeted interventions. Preclinical models using GLP-1–estrogen conjugates have shown superior outcomes in metabolic regulation, neuronal viability, and hypothalamic signaling, with reduced systemic estrogenic effects. These findings open a new frontier in personalized neuroendocrine therapy, particularly relevant for postmenopausal women, who bear a disproportionate burden of AD and are often underrepresented in clinical trials.

Future research must prioritize the inclusion of sex as a biological variable, explore differential receptor expression and function, and validate the safety and efficacy of dual agonist strategies in humans. As large-scale trials like EVOKE and EVOKE + unfold, integrating insights from estrogen biology could enhance the impact of GLP-1–based therapies in neurodegenerative disease. Ultimately, a deeper understanding of hormone–metabolism interactions may unlock novel, sex-specific strategies for the prevention of Alzheimer’s disease.
